# *UGT1A1* rs4148323 A Allele is Associated With Increased 2-Hydroxy Atorvastatin Formation and Higher Death Risk in Chinese Patients With Coronary Artery Disease

**DOI:** 10.3389/fphar.2021.586973

**Published:** 2021-03-08

**Authors:** He-Ping Lei, Min Qin, Li-Yun Cai, Hong Wu, Lan Tang, Ju-E Liu, Chun-Yu Deng, Yi-Bin Liu, Qian Zhu, Han-Ping Li, Wei Hu, Min Yang, Yi-Zhun Zhu, Shi-Long Zhong

**Affiliations:** ^1^ Research Center of Medical Sciences, Guangdong Provincial People’s Hospital, Guangdong Academy of Medical Sciences, Guangzhou, China; ^2^ Guangdong Provincial Key Laboratory of Coronary Heart Disease Prevention, Guangdong Cardiovascular Institute, Guangzhou, China; ^3^ School of Pharmacy, Macau University of Science and Technology, Macau, China; ^4^ School of Medicine, South China University of Technology, Guangzhou, China; ^5^ School of Pharmacy, Southern Medical University, Guangzhou, China; ^6^ Sun Yat-sen Memorial Hospital, Sun Yat-sen University, Guangzhou, China; ^7^ Department of Pharmacy, Guangdong Provincial People’s Hospital, Guangdong Academy of Medical Sciences, Guangzhou, China

**Keywords:** atorvastatin, coronary artery disease, *UGT1A1∗*6, polymorphisms, ADME genes, clinical outcomes

## Abstract

It is widely accepted that genetic polymorphisms impact atorvastatin (ATV) metabolism, clinical efficacy, and adverse events. The objectives of this study were to identify novel genetic variants influencing ATV metabolism and outcomes in Chinese patients with coronary artery disease (CAD). A total of 1079 CAD patients were enrolled and followed for 5 years. DNA from the blood and human liver tissue samples were genotyped using either Global Screening Array-24 v1.0 BeadChip or HumanOmniZhongHua-8 BeadChip. Concentrations of ATV and its metabolites in plasma and liver samples were determined using a verified ultra-performance liquid chromatography mass spectrometry (UPLC-MS/MS) method. The patients carrying A allele for the rs4148323 polymorphism (*UGT1A1*) showed an increase in 2-hydroxy ATV/ATV ratio (*p* = 1.69E−07, false discovery rate [FDR] = 8.66E−03) relative to the value in individuals without the variant allele. The result was further validated by an independent cohort comprising an additional 222 CAD patients (*p* = 1.08E−07). Moreover, the rs4148323 A allele was associated with an increased risk of death (hazard ratio [HR] 1.774; 95% confidence interval [CI], 1.031–3.052; *p* = 0.0198). In conclusion, our results suggested that the *UGT1A1* rs4148323 A allele was associated with increased 2-hydroxy ATV formation and was a significant death risk factor in Chinese patients with CAD.

## Introduction

Atorvastatin (ATV), which reduces low-density lipoprotein cholesterol (LDL-C) by inhibiting 3-hydroxy-3-methylglutaryl-coenzyme A (HMG-CoA) reductase, is among the most widely prescribed drugs for treating and preventing atherosclerotic disease events (Rosenson, 2006). The beneficial effects of ATV therapy in reducing the risk of cardiovascular morbidity and mortality have been well documented (Sever et al., 2003; Arca, 2007; Sillesen et al., 2008).

ATV is orally administered in the active acid form and is extensively metabolized by cytochrome P450 (CYP) 3A4 to form two major active metabolites, 2-hydroxy (2-OH) ATV and 4-hydroxy (4-OH) ATV (Park et al., 2008). Both metabolites are pharmacologically equivalent to parent ATV and significantly contribute to the circulating inhibitory activity for HMG-CoA reductase (Lennernas 2003). Glucuronidation, mediated via the enzymes UDP-glucuronosyltransferase (UGT) 1A1 and 1A3 (UGT1A1/3) in the liver, is the critical step in facilitating the conversion of the acid forms of ATV to the corresponding lactones (Prueksaritanont et al., 2002; Schirris et al., 2015). Thus, variations in the activities of drug metabolizing enzymes may result in lower or greater exposure to ATV.

Pharmacogenetic studies have shown that single-nucleotide polymorphisms (SNP) in genes related to absorption, distribution, metabolism and excretion (ADME) of drugs contribute to interindividual variability in drug efficacy and adverse effects (Lauschke et al., 2017; Guan et al., 2019). Failure to recognize these variants could lead to high systemic drug concentrations, which may increase rates of adverse events (Roden et al., 2019).

In this study, we focused particularly on the genes involved in ADME to identify novel genetic polymorphisms affecting plasma ATV and its metabolites concentrations and clinical outcomes of patients with coronary artery disease (CAD). Subsequently, we aimed to identify specific SNP associated with ATV metabolism in human liver microsomes (HLM).

## Methods

### Ethics Statement

This study was approved by the Medical Ethical Review Committee of Guangdong Provincial People's Hospital (Approval number GDREC2010137H) and Sun Yat-sen Memorial Hospital (Approval number CS07095) (Guangzhou, China), and conducted in accordance with the basic principles of the Declaration of Helsinki. All patients provided written informed consent.

### Study Population

**FIGURE 1:**
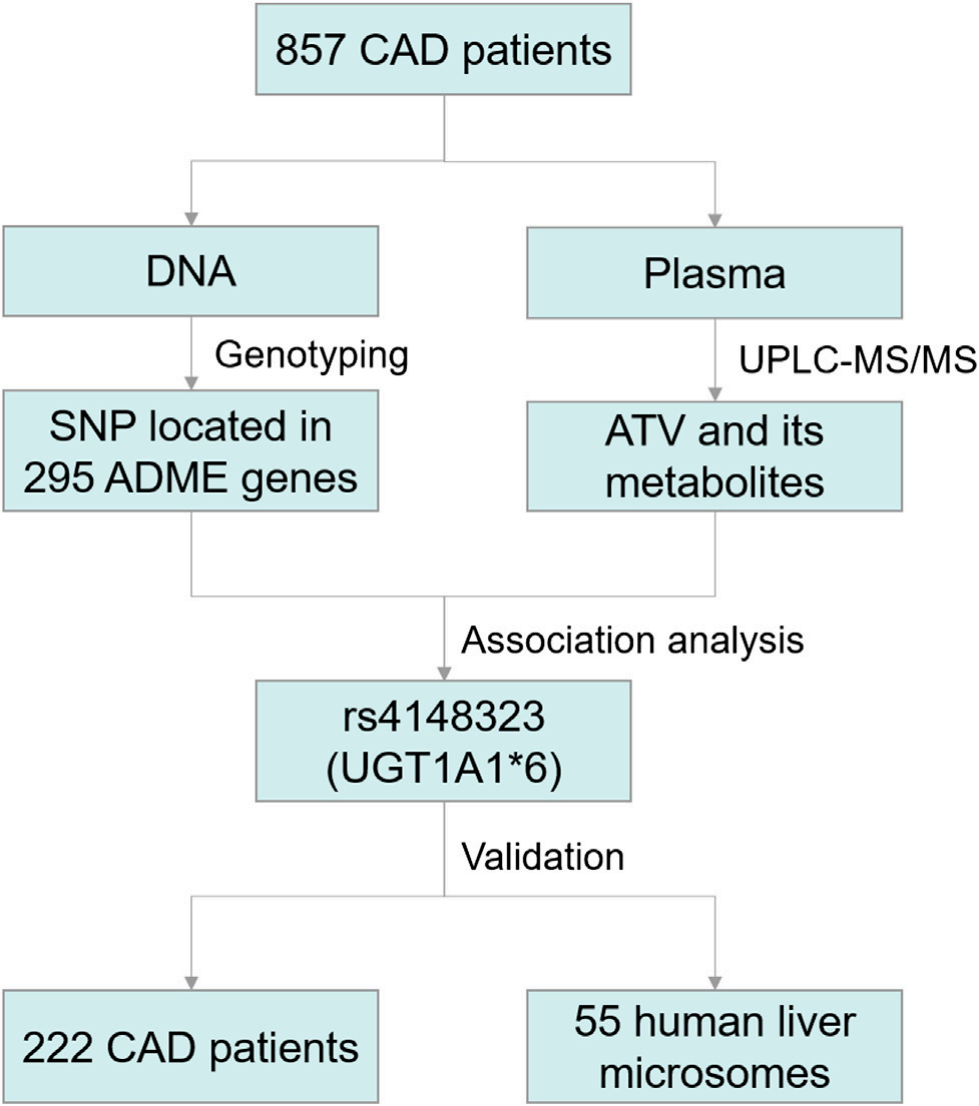
Schematic diagram of the Trial Protocol. CAD, coronary artery disease; ATV, atorvastatin; UGT1A1, UDP-glucuronosyltransferase 1A1; SNP, single-nucleotide polymorphisms; ADME, absorption, distribution, metabolism, and excretion.

A schematic diagram of this study was exposited in Figure 1. A total of 1079 CAD patients were categorized into two cohorts to discover and validate the effects of genetic variants on ATV metabolism and the risk of all-cause death. Thereafter, 55 HLM were enrolled to verify the effect of enzyme activity of *UGT1A1* on ATV metabolism and the correlation between UGT1A1*6 and the formation rate of 2-OH ATV. All patients were sequentially enrolled in Guangdong Provincial People's Hospital between January 2010 and December 2013 according to the inclusion and exclusion criteria. Patients were followed up for all-cause death up to 5 years. CAD was defined as the presence of ≥50% stenosis in at least one major coronary artery based on coronary angiography. The inclusion criteria were patients with CAD aged 18–80 years who underwent percutaneous coronary intervention (PCI) and received ATV therapy. Exclusion criteria included renal impairment (serum creatinine >3 times the upper limit of normal (ULN), renal transplantation or dialysis); liver impairment (serum transaminase >3 times the ULN, or a diagnosis of cirrhosis); pregnancy or lactation; malignant disease; uncontrolled infection; worsening of any chronic disease; use of lipid-lowering drugs other than ATV.

All patients received ATV for at least seven consecutive days at a dose of 10–40 mg/day before blood samples were collected. The dose of ATV was chosen based on the discretion of the physician. Steady-state ATV concentrations could be reached after approximately 3 days (Cilla et al., 1996). Baseline medical information was collected from the hospital medical records, including demographics, medical history, biochemical measurements, and comedications. Drug compliance was monitored by contacting with the patients at hospitalization or hospital visit. Patients were contacted every 6 months via telephone for surveillance of all-cause death. Individuals who could not be contacted despite several attempts were considered as lost to follow-up.

### Blood Sampling

Fasting venous blood (4 ml) was drawn into ethylenediaminetetraacetic acid (EDTA)-containing tubes 10–12 h after the last ATV dose. Samples were centrifuged 1900 *g* for 10 min at 4°C; plasma was collected and stored at −80°C until analysis.

### HLM Preparation

The tumor resection margin of patients with liver cancer or the liver tissues of patients with benign liver diseases undergoing hepatectomy were collected at Sun Yat-Sen Memorial Hospital (Guangzhou, China) from September 2012 to May 2015 (*n* = 55). Specimens for microsome extraction were quickly prepared using the GENMED A Solution (GENMED Scientific Inc., Arlington, TX, United States) and stored in liquid nitrogen until use. HLM were prepared according to our previously published protocol (Liu et al., 2016). Protein concentration was determined by the Bradford protein assay kit (Bio-Rad, Hercules, CA, United States) with bovine serum albumin as standard.

### Genotyping

Genomic DNA was extracted from blood samples using the TIANamp Genomic DNA Kit (Cat. no. DP304; TIANGEN Biotech, Beijing, China) per manufacturer's instructions. DNA quality and quantity were assessed using a NanoDrop 2000 spectrophotometer (Thermo Fisher Scientific, Waltham, MA, United States) and agarose gel electrophoresis, respectively.

In the discovery cohort, genotyping was performed for 857 DNA samples from patients with CAD on the Global Screening Array-24 v1.0 (GSA) BeadChip (Illumina Inc., San Diego, CA, United States) comprising 700,078 SNP. Genotyping procedures followed the Infinium HTS Assay protocol, and intensity data were normalized using Illumina's GenomeStudio software and calling algorithm. In the validation cohort comprising the other 222 patients with CAD, genotyping of *UGT1A1* c.211G > A (rs4148323) was performed by TaqMan assay (Applied Biosystems, California, United States). DNA from the human liver samples (*n* = 55) were genotyped using the HumanOmniZhongHua-8 BeadChip (Illumina Inc., San Diego, CA, United States) comprising 900,015 SNP.

A standard quality control procedure was applied to the raw genotyping data to filter both unqualified SNP and samples prior to association analysis. Samples with call rates <95% were removed. SNP were excluded if they 1) did not map on autosomal chromosomes; 2) had a call rate <95%; 3) had a minor allele frequency (MAF) < 5%; and 4) were deviated from Hardy–Weinberg equilibrium (*p*-value < 1.0E−06). After quality control, 291194 SNP in the GSA BeadChip and 695778 SNP in the HumanOmniZhongHua-8 BeadChip were retained for analysis.

### Determination of ATV and Its Metabolites Concentrations

Concentrations of ATV and its acid (2-OH ATV and 4-OH ATV) and lactone metabolites (ATV lactone [ATV L], 2-OH ATV L and 4-OH ATV L) in plasma were measured by ultra-performance liquid chromatography mass spectrometry (UPLC-MS/MS). Our previous report has established the accuracy and reproducibility of this method (Cai et al., 2017).

### Activity Determination of UGT1A1

The UGT1A1 activity in HLM was determined using the known substrate SN-38. The procedure was carried out based on our previously validated approach (Zhong et al., 2017).

### ATV Metabolism in HLM

A typical phase I and II enzymes mixing incubation system contains potassium phosphate buffer (50 mM, pH = 7.4), phase I Solution A and B, HLM (final concentration 0.35 mg/ml), ATV (final concentration 1.5 μg/ml), phase II Solution A and B in a total volume of 400 μL. Incubations were carried out for 60 min at 37°C in a shaking water bath. After the incubation, 60 µL ice-cold acetonitrile containing internal standard carbamazepine (100 ng/ml) were added to terminate the enzyme activity. All experiments were performed in triplicate. The samples were centrifuged at 15,000 *g* for 30 min at 4°C, and then ATV and its major metabolites in supernatant was analyzed by UPLC-MS/MS method as previously described (Cai et al., 2017).

### Statistical Analyses

Demographic and clinical characteristics were described as follows: continuous variables are presented as mean ± standard deviation (SD) and categorical variables are presented as counts (percentages). Normality was evaluated by the Shapiro–Wilk test. Natural-log transformation was performed prior to statistical analysis since the raw ATV analyte concentrations did not follow a normal distribution. Univariate linear regression analysis was used to assess the relationships between the baseline characteristics and plasma ATV concentration, and the significant characteristics (*p*-value < 0.05) were included into multivariate linear regression analysis.

In the discovery stage, SNP located in 295 candidate ADME genes from the PharmaADME website (http://www.pharmaadme.org/) were employed to association analysis. Chi-square test was used to estimate the Hardy-Weinberg equilibrium. Linear regression analysis under the additive mode was used to identify the associations between the candidate SNP and the concentrations of ATV, five metabolites (2-OH ATV, 4-OH ATV, ATV L, 2-OH ATV L and 4-OH ATV L) and five concentration ratios (2-OH ATV/ATV, 4-OH ATV/ATV, ATV L/ATV, 2-OH ATV L/ATV, 4-OH ATV L/ATV). In addition to sex, age and ATV dose, aspartate aminotransferase (AST) and creatinine (CREA) levels were also included for adjustment since they were significantly associated with plasma ATV concentration (Table 1). The linkage disequilibrium (LD) analyses were conducted to identify independent SNP between SNP pairs located in same chromosome and the *r* ^2^ of two SNP exceeding 0.5 was considered in LD. The false discovery rate (FDR) was used to correct the number of SNP and association analyses for multiple hypothesis testing. The significant correlation (*FDR* < 0.05) between SNP and metabolite concentration ratio was repeatedly investigated in the validation cohort. For SNP pairs in LD, only the SNP with the most significant *p* value was selected.

**TABLE 1:** 1,079 patient characteristics and their effects on plasma concentration of ATV.

Characteristics		Value *N* (%) or mean ± SD	Plasma ATV concentration, ng/mL			
			Univariable analysis		Multivariable analysis	
			Estimate	*p*-value	Estimate	*p*-value
Demographic data						
Total number		1,079				
Age (years)		62.95 ± 10.07	0.010	1.02E−02		
Sex	Female	218 (20.20)	0.029	7.63E−01		
	Male	861 (79.80)				
Dosage (mg)	10	19 (1.76)	0.017	2.13E−03	0.014	2.69E−02
	20	924 (85.63)				
	40	136 (12.60)				
Medical history						
Arrhythmia	No	984 (91.38)	−0.158	2.43E−01		
	Yes	93 (8.62)				
Diabetes	No	779 (72.33)	−0.014	8.70E−01		
	Yes	298 (27.67)				
Heart failure	No	986 (91.55)	−0.182	1.82E−01		
	Yes	91 (8.45)				
Hypertension	No	432 (40.07)	0.044	5.73E−01		
	Yes	646 (59.93)				
Hyperlipidemia	No	956 (88.68)	0.054	6.51E−01		
	Yes	122 (11.32)				
Biochemical measurements						
ALT, U/L		27.50 ± 13.37	0.010	1.13E−03		
AST, U/L		26.77 ± 11.29	0.020	6.44E−09	0.021	1.80E−04
CREA, umol/L		86.37 ± 24.87	0.006	2.99E−04	0.004	3.47E−04
eGFR, ml/min/1.73m^2^		94.24 ± 72.49	−0.001	8.50E−02		
CK, U/L		111.95 ± 110.51	0.000	4.07E−01		
CKMB, U/L		7.55 ± 6.03	0.001	9.20E−01		
CHOL, mmol/L		4.29 ± 1.13	0.093	6.44E−03		
LDL-C, mmol/L		2.59 ± 0.93	0.133	1.26E−03		
HDL-C, mmol/L		0.96 ± 0.26	−0.314	3.67E−02		
TRIG, mmol/L		1.61 ± 1.11	0.042	2.23E−01		
GLUC, mmol/L		6.73 ± 2.74	0.015	2.90E−01		
Lpa, mmol/L		303.24 ± 324.14	0.000	1.89E−01		
Apo (a), g/L		1.04 ± 0.27	−0.451	3.34E−03		
Medication						
β-blockers	No	114 (10.58)	0.023	8.52E−01		
	Yes	963 (89.42)				
ACEIs	No	390 (36.21)	−0.081	3.07E−01		
	Yes	687 (63.79)				
CCBs	No	775 (71.96)	0.096	2.56E−01		
	Yes	302 (28.04)				
PPI	No	552 (51.25)	0.085	2.65E−01		
	Yes	525 (48.75)				

Estimates were calculated by applying a linear regression model. Variables with *p* < 0.05 were included into the multivariable analysis. SD = standard deviation; ALT = alanine aminotransferase; AST = aspartate aminotransferase; CK = creatine kinase; eGFR = estimated glomerular filtration rate; CKMB = creatine kinase MB; CHOL = cholesterol; LDL-C = low density lipoprotein cholesterol; HDL-C = high density lipoprotein cholesterol; TRIG = triglyceride; GLUC = glucose; Lpa = lipoprotein (a); Apo (a) = apolipoprotein (a); ACEIs = angiotensin converting enzyme inhibitors; CCBs = calcium channel blockers; PPIs = proton pump inhibitors.

Spearman correlation analysis was used to study the correlation between the UGT1A1 enzyme activity and the reduction of ATV as well as the formation rate of its five metabolites. To examine relations between the candidate SNP and the reduction of ATV as well as the formation of metabolites from ATV in 55 HLM, the independent sample *t* test or one-way ANOVA test was used for data conforming to normal distribution, whereas the nonparametric Mann–Whitney *U* or Kruskal–Wallis *H* test was used for data conforming to skewed distribution. Cox regression analysis was utilized to assess the association of SNP with all-cause death with results presented as hazard ratio (HR) and 95% confidence interval (CI). Cumulative event rates were estimated with the Kaplan–Meier method. A two-sided *p-*value < 0.05 was considered statistically significant.

All statistical analyses were carried out using PLINK (version 1.07, http://zzz.bwh.harvard.edu/plink/), R (version 3.4.3, https://www.r-project.org/) and GraphPad Prism 8 (GraphPad Software, San Diego, CA, United States).

## Results

### Patient Characteristics and Their Effects on Plasma ATV Concentrations

Patients' demographic and clinical characteristics and their impact on the plasma ATV concentrations are presented in Table 1. In total, 1,079 Chinese patients with CAD who had received ATV therapy were sequentially recruited in the study and followed for 5 years. Univariate linear regression analysis indicated that patients with older age, higher ATV dose, higher levels of alanine aminotransferase (ALT), AST, CREA, CHOL and LDL-C tended to have a higher plasma ATV concentration, while patients with higher levels of high-density lipoprotein cholesterol (HDL-C) and apolipoprotein (a) [Apo (a)] tended to have a lower plasma ATV concentration. In the multivariate model, only ATV dose, AST and CREA levels remained independent predictors of plasma ATV concentrations (*p* = 2.69E−02, 1.80E−04 and 3.47E−04, respectively) in CAD patients (Table 1).

### rs4148323 was Associated With Higher Concentration Ratio of 2-OH ATV to ATV

**FIGURE 2:**
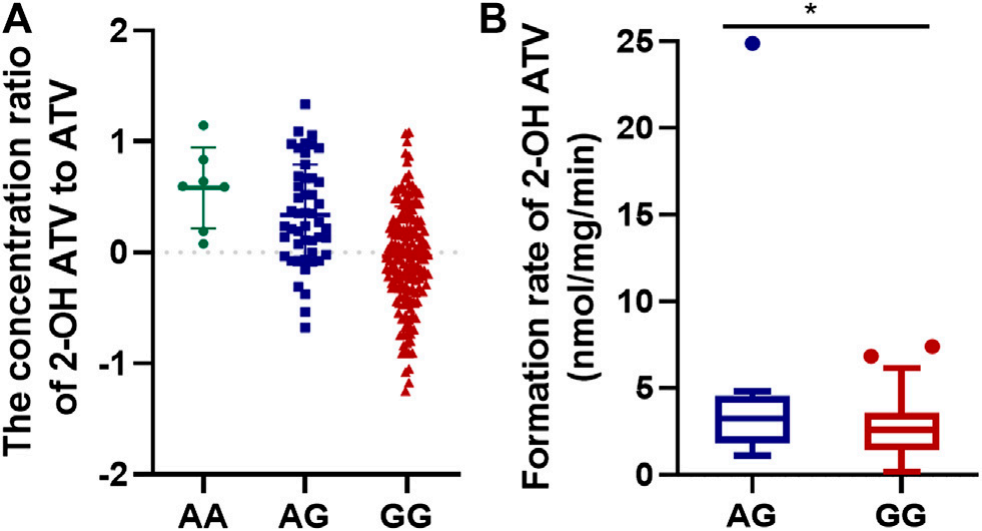
The effect of genotype of rs4148323 on the formation of 2-OH ATV in the 222 patients with CAD **(A)** and 55 HLM **(B)**. ATV, atorvastatin; CAD, coronary artery disease; HLM, human liver microsomes.

Ten SNP were found to have a significant effect on the concentration ratio of 2-ATV to ATV (*FDR* < 0.05, Table 2). Among these SNP, an exonic variant of rs4148323 in *UGT1A1* was most strongly associated with an increase in the 2-OH ATV/ATV ratio. Five SNP (rs15524, rs4646457, rs4646450, rs776746 and rs4646458) in *CYP3A5* also showed significant positive correlations with the formation of 2-OH ATV. Furthermore, an intergenic variant (rs10242455 between *ZSCAN25* and *CYP3A5*) and three intronic variants (rs2687136 and rs2687134 in *CYP3A7*; rs3806598 in *UGT1A10*) were also significantly associated with 2-OH ATV/ATV ratio (Table 2). Further analysis indicated that rs4148323 was in strong LD with rs3806598, and rs15524 was in strong LD with the remaining seven loci located in chromosome 7 (*r* ^2^ > 0.5). Finally, rs4148323 was further verified to be significantly correlated with 2-OH ATV/ATV ratio in an independent cohort comprising an additional 222 CAD patients (*p* = 1.08E−07, Figure 2A).

**TABLE 2:** Ten SNPs significantly associated with the concentration ratio of 2-OH ATV to ATV in 857 patients with CAD.

SNP	CHR	BP	Change	Gene symble	Ref	Alt	2-OH ATV/ATV		
							Beta	*P*	FDR
rs4148323	2	234669144	Exonic	UGT1A1	G	A	0.184	1.69E−07	8.66E−03
rs15524	7	99245914	UTR3	CYP3A5	A	G	0.129	8.52E−07	1.09E−02
rs10242455	7	99240179	Intergenic	ZSCAN25, CYP3A5	A	G	0.129	8.52E−07	1.09E−02
rs4646457	7	99245080	Downstream	CYP3A5	A	C	0.129	8.52E−07	1.09E−02
rs2687136	7	99325882	Intronic	CYP3A7, CYP3A7-CYP3A51P	C	T	0.125	2.23E−06	1.63E−02
rs2687134	7	99331042	Intronic	CYP3A7, CYP3A7-CYP3A51P	T	G	0.124	2.29E−06	1.63E−02
rs4646450	7	99266318	Intronic	CYP3A5	G	A	0.124	2.60E−06	1.63E−02
rs776746	7	99270539	Splicing	CYP3A5	C	T	0.124	2.86E−06	1.63E−02
rs4646458	7	99245013	Downstream	CYP3A5	T	G	0.124	5.27E−06	2.70E−02
rs3806598	2	234579892	Intronic	UGT1A10, UGT1A8	A	C	0.151	7.2E−06	3.35E−02

Ref reference allele, Alt alternate allele, UTR untranslated region, SNP, single-nucleotide polymorphisms; ATV, atorvastatin; 2-OH ATV, 2-hydroxy atorvastatin; CHR, chromosome; BP, base position; FDR, false discovery rate.

The p-values were calculated based on the linear regression analysis under the additive mode. The FDR were calculated on the basis of Benjaminiand Hochberg method. The SNPs are annotated to the nearest gene if identified in this study (marked by asterisk symbol) or to previously known gene if in linkage disequilibrium with the known loci for any lipid measure. Chromosomal positions are based on hg19 reference sequence.

### Influence of the Genotype of rs4148323 on the Formation Rate of 2-OH ATV in HLM

To verify the effect of rs4148323 on the rates of formation of 2-OH ATV from ATV, the association between genotypes and 2-OH ATV formation rate was investigated in 55 HLM. The results showed that SNP rs4148323 in *UGT1A1* was associated with changes in 2-OH ATV levels (5.30 ± 7.44 and 2.71 ± 1.68 nmol/mg/min for AG and GG, respectively; *p* = 0.026, Figure 2B).

### The Correlation Between UGT1A1 Activity and the Metabolism of ATV in HLM

**FIGURE 3:**
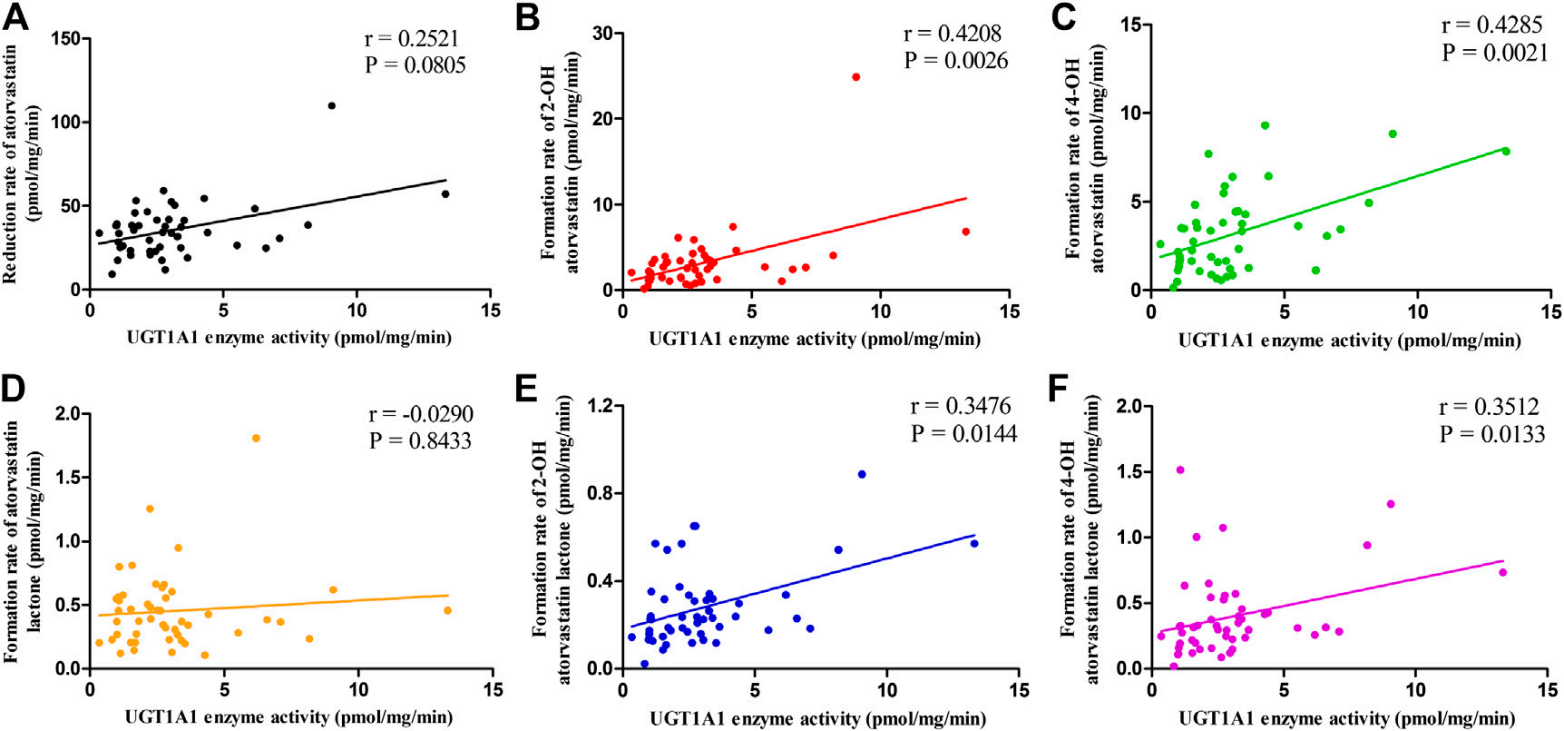
The correlations between UGT1A1 activity and the rates of microsomal metabolism of ATV **(A)**, 2-OH ATV **(B)**, 4-OH ATV **(C)**, ATV L **(D)**, 2-OH ATV L **(E)** and 4-OH ATV L **(F)**. UGT1A1, UDP-glucuronosyltransferase 1A1; ATV, atorvastatin; ATV L, atorvastatin lactone.

Correlation between UGT1A1 activity and rates of microsomal metabolism of ATV and its metabolites are detailed in Figure 3. Higher UGT1A1 activity was associated with a markedly increased formation rates of 2-OH ATV, 4-OH ATV, 2-OH ATV L and 4-OH ATV L (*r* = 0.4208, *p* = 0.0026; *r* = 0.4285, *p* = 0.0021; *r* = 0.3476, *p* = 0.0144; *r* = 0.3512, *p* = 0.0133). In contrast, the activity of UGT1A1 was not correlated with the reduction rate of ATV and the formation rate of ATV L (*p* = 0.0805 and 0.8433, respectively) (Figure 3).

### Impact of Genetic Polymorphisms on the Clinical Endpoint

**FIGURE 4:**
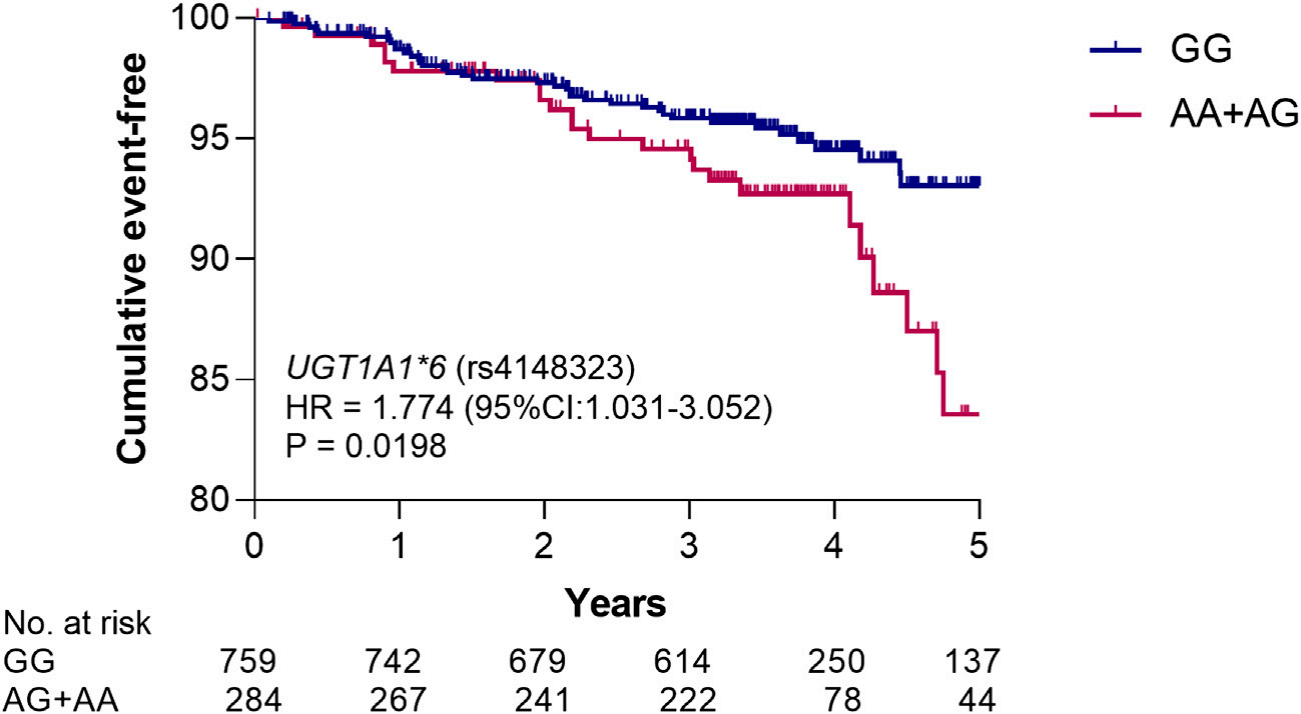
Kaplan–Meier analysis of rs4148323 for all-cause death risk. The *p* value was calculated by log-rank test. UGT1A1, UDP-glucuronosyltransferase 1A1; HR, hazard ratio; 95% CI, 95% confidence interval.

In order to illustrate the genotype of rs4148323 whether has an effect on the poor prognosis of patients with CAD, we merged the discovery and validation cohorts to assess the association between genotypes and death risk. Due to the small number of patients with the AA genotype of rs4148323 (*n* = 16), the AG and AA individuals were grouped together into the AG + AA genotype group (the A allele carriers), for analysis. Kaplan-Meier survival analysis showed that the carriers of rs4148323 A allele have a higher risk of death than non-carriers (HR 1.774, 95% CI, 1.031–3.052; *p*  = 0.0198) (Figure 4).

## Discussion

Our result showed that a variant of rs4148323, located in an *UGT1A1* exon, increased the plasma ATV's active metabolite 2-OH ATV formation. This finding was further validated by an independent cohort comprising an additional 222 CAD patients and by the human liver microsome systems. Furthermore, the *UGT1A1* rs4148323 A allele has a significantly higher risk of death in Chinese patients with CAD. Consequently, genotyping of rs4148323 might be useful for tailoring both the ATV dose and safety monitoring of CAD patients.

Despite tremendous progress due to lifestyle interventions and drug treatments, CAD remains one of the most significant cause of death worldwide (Georgia Karanasiou et al., 2018). ATV is a life-saving drug which leads to reduce cardiovascular events in patients with cardiovascular disease, providing substantial public health benefits (Crouch 2001). ATV exists in both the acid and lactone forms *in vivo*. The acid form is pharmacologically active, whereas the lactone form is inactive toward HMG-CoA reductase and has been associated with muscle-related adverse effects (Hermann et al., 2006; Skottheim et al., 2008). ATV-induced liver injury can be caused during ATV therapy. The higher hepatocellular concentration of ATV was found to increase the risk of hepatotoxicity since ATV induced cytotoxicity in HepaRG cells in a concentration-dependent manner (Fukunaga et al., 2016). We have previously shown that high plasma exposure of statins was associated with an increased risk of contrast-induced acute kidney injury in patients with CAD; therefore, statins should be used with caution in these patients (Cai et al., 2018). We also found that a higher plasma exposure of ATV and metabolites was linked to increased risk of death in CAD patients (Zhou et al., 2020).

Interindividual differences in efficacy of ATV may be caused not only by nongenetic factors, but also by genetic polymorphisms in drug metabolizing enzymes and transporters involved in ATV metabolism and elimination (Kivisto et al., 2004; Cho et al., 2012; Wei and Zhang 2015; Peng et al., 2018). UGT1A1 is an important member of the UGT1A family responsible for the conjugation and detoxification of numerous endogenous and exogenous compounds (Levesque et al., 2007). Defects in this enzyme result in unconjugated hyperbilirubinemia, such as Gilbert syndrome and Crigler–Najjar syndrome (Kadakol et al., 2000). The genetic polymorphism UGT1A1*6 (rs4148323, c.211G > A, Arg71Gly) is an exonic variant of the *UGT1A1* gene on chromosome 2q37 and associated with reduced UGT1A1 activity (Bai et al., 2019). UGT1A1*6 is highly prevalent in East Asian populations but is absent in European and African populations (Dai et al., 2013). It has allele frequencies of 23%, 23%, 13%, and 0% among Chinese, Korean, Japanese, and German populations, respectively (Akaba et al., 1998). It was reported that one of the metabolic pathways of ATV is through UGT1A1-mediated glucuronidation (Schirris et al., 2015) and the A allele in *UGT1A1* rs4148323 is associated with decreased UGT1A1 activity (Bai et al., 2019). Therefore, we speculated that the rs4148323 A allele might decrease glucuronidation activity for ATV and corresponding increase in 2-OH ATV production.

Many studies have reported genetic variants were associated with CAD pathogenesis (McPherson and Tybjaerg-Hansen 2016; Miao et al., 2018). Despite an enormous amount of research that has been done on the biological effect of *UGT1A1* gene (Goon et al., 2016), few studies have assessed whether the rs4148323 SNP has a prognostic value on all-cause death among CAD patients. To our knowledge, we are the first to demonstrate that the rs4148323 A allele was associated with increased risk of death in CAD patients.

CYP3A5 is an important hepatic drug-metabolizing enzyme. Willrich *et al.* found that the CYP3A5*3A allele was associated with reduced cholesterol-lowering response to ATV in 139 non-African individuals with hypercholesterolemia (Willrich et al., 2008). In the present study, positive correlations were found between SNP (rs15524, rs4646457, rs4646450, rs776746 and rs4646458) in the *CYP3A5* gene and the formation of 2-OH ATV. ATV and its active metabolites are subject to cellular membrane transport by organic anion-transporting polypeptide (OATP) transporters and P-glycoprotein (P-gp) (Bogman et al., 2001; Chen et al., 2005). Despite evidence that drug transporter polymorphisms could influence ATV metabolism (Lee et al., 2010; Wang et al., 2017), we did not observe such an association *in vivo* and the reason for this result is unclear.

Our study had two limitations. First, the study subjects were primarily Han ethnic Chinese, and that caution may be warranted in extrapolating our results to other populations. Second, the sample size was relatively small. In order to minimize the finding of false positive statistical associations, the *p* values were adjusted using the FDR.

In summary, the *UGT1A1* rs4148323 A allele was found to be significantly associated with elevated 2-OH ATV formation, and might increase the risk of death in Chinese patients with CAD. The present study provides suggestive data, and genotyping large cohorts of CAD patients for rs4148323 in *UGT1A1* gene will be required to unambiguously prove these findings.

## Data Availability

The datasets presented in this study can be found in online repositories. The names of the repository/repositories and accession number(s) can be found below: EMBL-EBI [Project: PRJEB42554; Analyses: ERZ1714343].

## Glossary

ATV: atorvastatin
GLUC: glucose
TRIG: triglycerides
CHOL: cholesterol
HDL-C: high-density lipoprotein cholesterol
LDL-C: low-density lipoprotein cholesterol
CREA: creatinine
ALT: alanine aminotransferase
AST: aspartate aminotransferase
CK: creatine kinase
CKMB: creatine kinase MB
Lp (a): lipoprotein (a)
Apo (a): apolipoprotein (a)
CAD: coronary artery disease
FDR: false discovery rate
ADME: absorption, distribution, metabolism, and excretion
CYP: cytochrome P450
HLM: human liver microsomes
HR: hazard ratio
OR: odds ratio
CI: confidence interval
eGFR: estimated glomerular filtration rate
MAF: minor allele frequency
PCI: percutaneous coronary intervention
CCB: calcium channel blocker
ACEI: angiotensin-converting enzyme inhibitor
PPI: proton pump inhibitor
SNP: single-nucleotide polymorphisms
LD: linkage disequilibrium
UGT: UDP-glucuronosyltransferase
ULN: upper limit of normal
SD: standard deviation
UPLC-MS/MS: ultra-performance liquid chromatography mass spectrometry
HMG-CoA: 3-hydroxy-3-methylglutaryl-coenzyme A
OATP: organic anion transporting polypeptide
P-gp: P-glycoprotein
